# A Class-Independent Texture-Separation Method Based on a Pixel-Wise Binary Classification

**DOI:** 10.3390/s20185432

**Published:** 2020-09-22

**Authors:** Lucas de Assis Soares, Klaus Fabian Côco, Patrick Marques Ciarelli, Evandro Ottoni Teatini Salles

**Affiliations:** 1 Federal Institute of Espírito Santo, Linhares 29901-291, Brazil; 2 Department of Electrical Engineering, Federal University of Espírito Santo, Vitória 29075-910, Brazil; klaus.coco@ufes.br (K.F.C.); patrick.ciarelli@ufes.br (P.M.C.); evandro.salles@ufes.br (E.O.T.S.)

**Keywords:** convolutional neural networks, texture analysis, texture separation

## Abstract

Texture segmentation is a challenging problem in computer vision due to the subjective nature of textures, the variability in which they occur in images, their dependence on scale and illumination variation, and the lack of a precise definition in the literature. This paper proposes a method to segment textures through a binary pixel-wise classification, thereby without the need for a predefined number of textures classes. Using a convolutional neural network, with an encoder–decoder architecture, each pixel is classified as being inside an internal texture region or in a border between two different textures. The network is trained using the Prague Texture Segmentation Datagenerator and Benchmark and tested using the same dataset, besides the Brodatz textures dataset, and the Describable Texture Dataset. The method is also evaluated on the separation of regions in images from different applications, namely remote sensing images and H&E-stained tissue images. It is shown that the method has a good performance on different test sets, can precisely identify borders between texture regions and does not suffer from over-segmentation.

## 1. Introduction

Textures constitute an important feature for human visual discrimination [1]. Even though they do not have a precise definition in the literature, they are normally characterized by their pattern, regularity, granularity, and complexity [2]. Although the segmentation of textures has direct applications in medical image analysis [3,4,5,6,7] quality inspection [8,9], content-based image retrieval [10], analysis of satellite and aerial images [11,12,13], analysis of synthetic aperture sonar images [14], and object recognition [15], it is still a challenging problem due to its subjective nature and variability in terms of viewpoints, scales, and illumination conditions [16].

Texture analysis methods in digital images can be broadly divided into four categories [17,18,19]: statistical methods, structural methods, model-based methods, and filter-based methods. Statistical methods describe textures based on local spatial distribution of pixel intensities using statistical measures. As a classical example of statistical method in texture analysis, the gray level co-occurrence matrix technique [20,21] uses local statistics based on the spatial relationship between neighboring pixels in the image. This technique has originated many variations [22,23,24,25] and is widely used in applications of texture classification [26,27]. Another example of a statistical approach in texture analysis is the local binary patterns (LBP) [28], and its variations [29,30,31], which analyze the texture content of an image by comparing pixels and their neighbors by searching for local patterns is also used in many works that deal with texture classification [32,33].

Structural methods describe textures using well-defined primitives and the spatial relationship between those primitives using placement rules [21]. Those primitives are considered to be fundamental structures of textural visual perception, known in the literature as textons [34,35]. Another structural approach based on morphological mathematical operations is presented in [36]. Model-based techniques represent textures using stochastic models or generative image models. Examples of models used for describing textures are Markov random field models [37,38] and fractal models [39,40]. Finally, filter-based methods, also known as transform-based methods, describe textures through frequency analysis based on Fourier transforms [41,42], Gabor filters [43,44,45] and wavelets [46,47,48].

In the last few years, deep learning techniques and, more specifically, convolutional neural networks have presented remarkable results in computer vision applications [49,50,51,52,53] and naturally, in view of the significant results, these methods were also applied in image texture analysis. However, even though many applications related to textures in computer vision involving deep learning deal with texture classification [54,55,56,57,58,59,60,61,62] and texture synthesis [63,64,65], only a few papers present deep learning techniques applied to texture segmentation. In [66], a two-dimensional long short-term memory (LSTM) network was proposed to classify each pixel of an image according to a predefined class, where the spatial recurrent behavior of the network made it possible to consider neighborhood contributions to the final decision. A convolutional neural network was used in [62] to segment image patches found by a region proposal algorithm through their classification. The network was used as a feature extractor and its output was used in a Fisher Vector (FV) encoding followed by a support vector machine (SVM) classifier.

In [67], Andrearczyk and Whelan proposed a convolutional neural network with skip connections based on the fully convolutional network (FCN) [68] to combine information from shallower and deeper layers to segment textures. In [69], Huang et al. used a similar architecture, but they also employed texture features extracted from the images by using an empirical curvelet transform. Currently, this technique presents the best results on the Prague Texture Segmentation Datagenerator and Benchmark [70]. Although the results presented in [67,69] showed a good performance, the methods were restricted to a limited number of classes of textures in the images to perform the pixel-wise classification. Karabağ et al. [71] presented an evaluation on texture segmentation comparing a U-Net architecture with traditional algorithms, namely co-occurrence matrices, watershed method, local binary patterns, filters, and multi-resolution sub-band filtering.

Additionally, in [72], a Siamese convolutional neural network for texture feature extraction followed by a hierarchical region merging is proposed for unsupervised texture segmentation; and in [73], a convolutional neural network is used for one-shot texture segmentation, where a patch of texture is used to segment a whole region in another image through an encoder–decoder architecture.

This paper presents a novel method for detecting the edges between textures without the need for a prior knowledge about the types and number of texture regions in the image. Through an encoder–decoder architecture with skip connections, each pixel in the image is classified as being inside an internal texture region. In this paper, the term internal texture region refers to the internal part of a texture region of the image or in a border between two or more different textures. This way, the technique is not restricted to a specific number of texture classes and it is able to segment images with textures classes that were not present in the training stage. Moreover, the method does not need any pre-processing step and is able to separate a texture mosaic directly from the original image.

This approach is different from other pixel classification segmentation approaches in which each pixel is classified as a predefined class from a limited prior number of classes, such as the popular U-Net [74] and SegNet [75], and the texture segmentation networks proposed by Andrearczyk and Whelan (2017) [67] and Huang et al. (2019) [69].

The proposed technique is also different from an edge detector because it detects edges with a highly specific purpose. The only edges detected by the presented approach are supposed to be the boundaries between two or more textures, whereas, on the other hand, the edges in an internal texture region should be ignored, despite the internal contrast that the texture may present.

The paper is structured as follows. Section 2 describes the neural network architecture in detail. Section 3 presents the datasets used in the experiments. Section 4 shows the results comparing the segmentation with a conventional class-dependent convolutional neural network method and the performance of the proposed method tested on different texture sets, on mosaics containing remote sensing images and on Hematoxylin and Eosin-stained tissue images. Section 5 concludes the paper.

## 2. Neural Network Architecture

In the presented method, a convolutional neural network is used to identify the boundaries between different texture regions in an image. The convolutional neural network has an encoder–decoder architecture with skip connections that is similar to other well-known architectures in the literature, such as the U-Net [74] and SegNet [75]. This architecture is used because it can output an image of the same dimensions as the input image. The encoder part is responsible for mapping the main features that help on the identification of borders between different textures in the mosaic image, but due to the max pooling layers, it reduces the input dimensions. The decoder part is then responsible for retrieving the original dimensions of the image using the information from the encoder part. Moreover, the skip connections help this process by using information from different layers.

**Figure 1:**
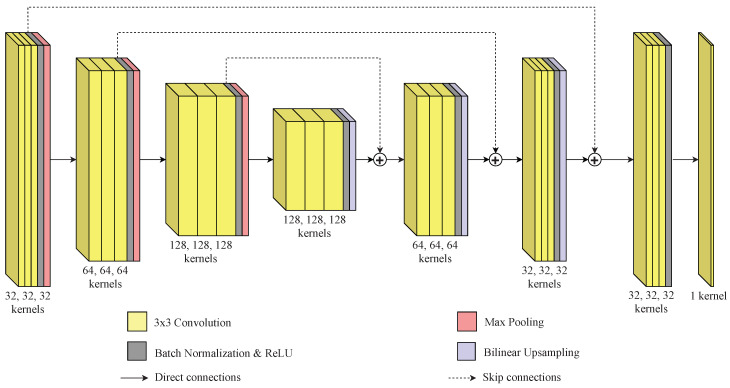
Network architecture.

This paper proposes to perform the training process in an end-to-end manner considering images depicting boundaries between different texture regions. Figure 1 presents the network architecture. The first three blocks are the compression blocks (the encoder part of the network), in which the weights are expected to be adjusted to suppress internal borders in texture regions while preserving the borders between different texture regions as the information is propagated to the deeper layers. This weight adjustment is performed by analyzing patches of the input image, given by the convolutional kernels in different scales which, in turn, are given by the different layers of the network.

Following the encoder part, the expansion (or decoder) part of the network is composed by three blocks and is responsible for recovering the information from the receptive fields in the contraction blocks in order to produce an image with the same dimensions as the input image. In addition, skip connections between the contraction part and the expansion part are used so that the network can use information from different levels, combining the information of different layers to produce the output. Finally, a final processing block and an output layer form the last part of the network.

Each compression block is composed of three convolutional layers, a batch normalization layer which reduces the internal covariate shift [76] and accelerates the training [77], a rectified linear unit (ReLU) activation function [78] layer, and a maximum pooling layer that reduces the size of the input by half. The number of filters in the convolutional layers of each compression block is 32, 64, and 128, respectively.

The expansion blocks follow the same structure of three convolutional layers, a batch normalization layer, and a ReLU layer. However, instead of a maximum pooling layer, they have an upsampling layer that duplicates the size of the input using a bilinear interpolation function. The first expansion block follows the last compression block such that it is directly connected to its corresponding receptive field dimension at the compression step. The two last expansion layers combine the information from their previous expansion layer with the output of its corresponding receptive field dimension at the compression block, before the maximum pooling operation, through the sum of the filters. The number of filters in the convolutional layers of each expansion layer is 128, 64, and 32, respectively.

The input of the final processing layer block is the sum of the output of the last expansion block with the output of the first compression block before the maximum pooling operation. This block is formed by three convolutional layers with 32 filters each, a batch normalization layer, and a ReLU layer. Finally, at the end of the network there is an output layer with a single convolutional layer and a sigmoid activation function, since the model performs a binary classification. The input of this layer is the output of the final processing block. All the convolutional filters have the same size of 3×3. Table 1 summarizes the parameters of each layer.

It is worth noting that the proposed architecture is shallower than similar segmentation architectures. This is because the technique deals with textures instead of objects which are better represented by shapes whose information is represented in the deeper layers of convolutional neural networks [6].

For the training process, the objective function, *J*, for this binary pixel classification is defined by the binary cross-entropy error [79], described as:

(1) $$J=-\sum_{n=1}^{N}t_{n}\log\left(y_{n}\right)+\left(1-t_{n}\right)\log\left(1-y_{n}\right),$$

where *N* is the total number of samples, $t_{n}$ is the target class and $y_{n}$ is the predicted class. For a given sample, only one of the two terms, $t_{n}\log\left(y_{n}\right)$ or $\left(1-t_{n}\right)\log\left(1-y_{n}\right)$, in the summation is computed, since $t_{n}\in \left\{0,1\right\}$, and a perfect classification does not increase the cross-entropy error, because, in this case, $y_{n}=1$, if $t_{n}=1$, or $y_{n}=0$, if $t_{n}=0$, and the logarithm of the term being computed would be equal to zero.

The adjustment of the weights is performed using the Adam optimization algorithm [80] due to its popularity and performance in recent deep learning applications, but other gradient descent algorithms or other optimization techniques could be used as well. The convolutional neural network was programmed using a TensorFlow API [81] and the parameters used for the optimization algorithm were the default ones.

## 3. Texture Images Datasets

The texture images used in this paper are extracted from the Prague Texture Segmentation Datagenerator and Benchmark dataset [70], the Brodatz textures dataset [82], and the Describable Textures Dataset (DTD) [83].

**Figure 2:**
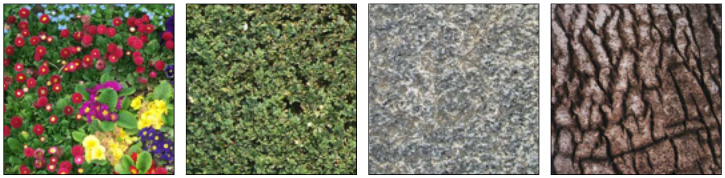
Texture images from the Prague Texture Segmentation Datagenerator and Benchmark [70].

The Prague Texture Segmentation Datagenerator and Benchmark dataset [70] has 114 color texture images from 10 thematic classes composed of natural and artificial textures, where each image contains a unique texture. Figure 2 presents some of the texture images from this dataset.

**Figure 3:**
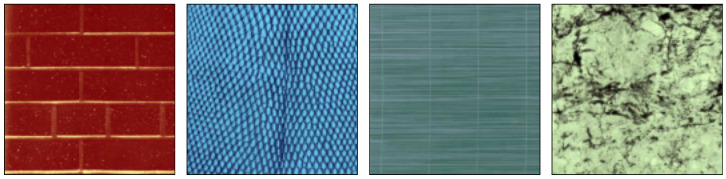
Texture images from the Brodatz dataset [82].

The Brodatz textures dataset [82] has 112 color texture images that are relatively distinct from each other and do not have much variation in illumination, scale, and rotation. Figure 3 show some of the texture images from this dataset.

**Figure 4:**
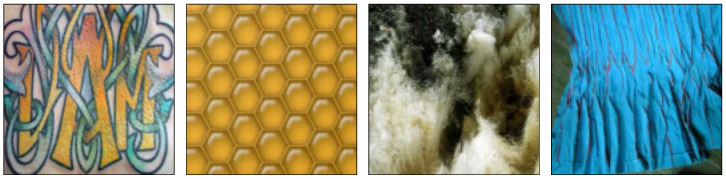
Texture images from the Describable Textures Dataset [83].

The Describable Textures Dataset [83] is a larger image set with 5640 different texture images. Those textures are more challenging than the ones from the other two datasets since there is significant variation in illumination, scale, and rotation in the same image. There is also a greater number of complex images, such as faces and natural scenes. Figure 4 presents some of the texture images from this dataset.

For the training, only 103 texture images from the Prague Texture Segmentation Datagenerator and Benchmark dataset were used for the creation of the texture mosaics. The other 11 texture images were used exclusively on the test mosaics. Moreover, texture images from the Brodatz dataset and from the Describable Textures Dataset were exclusively used for the tests.

Three different types of mosaic structures types were used: Voronoi mosaics, random walk mosaics, and circular mosaics. Regarding the Voronoi structure, the mosaic images were created by placing 2 to 10 centroids in random points in the image. Afterwards, each pixel of the image was assigned to a class according to the closest centroid. The random walk mosaics were created by choosing two random points, one in the horizontal borders and another one in the vertical borders of the image and performing a random walk in the opposite direction. Finally, the circular mosaics were created by placing up to four circles on the image with random centers and radius.

**Figure 5:**
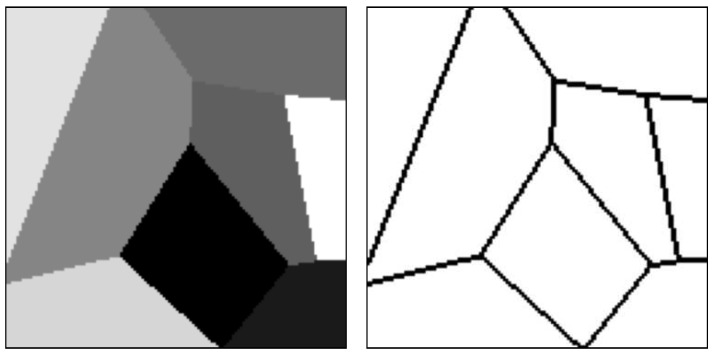
Example of mosaic structure and its associated borders.

**Figure 6:**
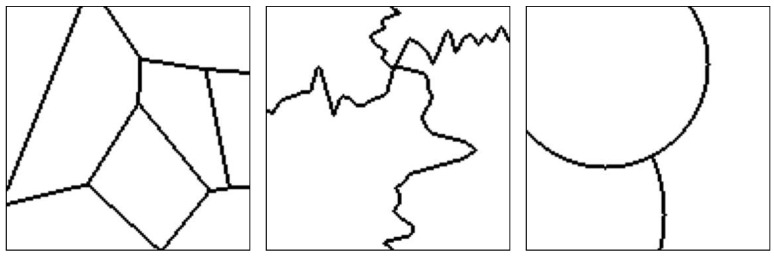
Example of mosaic structure and its associated borders.

All pixels placed in a transition between two or more different regions were labeled as being in a border between two textures, therefore, the borders in the targets are always more than two pixels wide. Figure 5 presents one mosaic structure with the different regions and the associated borders, and Figure 6 presents one of each type of mosaic structure.

For each mosaic image from the training set, a random type of structure was chosen and after the mosaic structure was created, each region was filled with a randomly chosen image from the 103 texture images belonging to the Prague Texture Segmentation Datagenerator and Benchmark dataset. In order to increase the network robustness, an image augmentation technique was applied to each texture [84].

The augmentation was made by randomly rotating, translating, shearing, and changing the scale of the image, applying a random uniform noise and a random image processing technique. The image processing techniques considered were adaptive histogram equalization, histogram equalization, logarithmic adjustment, sigmoid adjustment, a random Gamma correction, a random Gaussian blurring, or an image inversion [85]. All random parameters used for the image augmentation procedure were taken from uniform distributions. Table 2 presents the interval limits for each procedure. The techniques of adaptive histogram equalization, histogram equalization, logarithmic adjustment, and sigmoid adjustment used the default parameters of the scikit-image library [86].

With this procedure, 100,000 different training mosaics were created. Four different test sets were created with 10,000 mosaics each. The first one contained textures from the 103 images belonging to the Prague Texture Segmentation Datagenerator and Benchmark that were also used to construct the training mosaics, whereas the second test set had only textures from the 11 remaining texture images, from this dataset, that were not used for the training. The third and fourth test sets were filled with texture images from the Brodatz and Describable Textures Dataset, respectively. It is important to note that each individual mosaic image from the training and test sets had a different mosaic structure. This was done in order to prevent the network from learning the mosaic structure instead of the separation of textures.

## 4. Results

This section is divided in five subsections. In Section 4.1, a visual presentation of the results on a few examples of mosaic images is made in order to visually explain the results of the method and its advantages over a multi-label pixel classification technique and an edge detection method. In Section 4.2, the results of the method over the four different test sets are presented. In Section 4.3, a comparison with state-of-the-art methods on the Prague Texture Datagenerator and Benchmark is made. Finally, in Section 4.4 and Section 4.5, the technique is qualitatively evaluated on remote sensing and tissue images, respectively.

### 4.1. Visual Results on Test Mosaics

To perform a visual comparison of the results obtained by the proposed technique, the same convolutional encoder–decoder neural network architecture was trained, but instead of classifying each pixel in the image as being in a border between textures or in an internal texture region, each pixel was classified as a specific texture class in a similar manner as a fully convolutional network [67].

A comparison was also made with a popular edge detector called holistically nested edge detector (HED) [87]. In the original paper [87] the network of HED was defined considering the architecture of a VGG-16 [88] with the weights adjusted for the ImageNet dataset [89]. The network was then fine-tuned using the Berkeley Segmentation Dataset - BSDS 500 [90]. In this paper, the HED was initialized as in the original paper, but it was then trained with the same texture mosaics used for the training of the proposed method. This was made in order to show that a complex technique like HED results in over-segmentation, when employed for textures.

**Figure 7:**
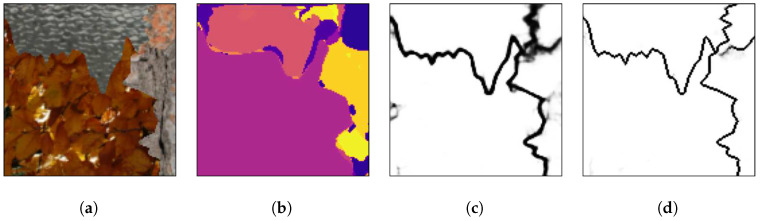
(**a**) An example mosaic, (**b**) the results of a multi-class labeling approach, (**c**) a HED architecture, and (**d**) the proposed method.

Figure 7 presents an arbitrary mosaic from the first test set, which has textures images from the Prague Texture Segmentation Datagenerator and Benchmark dataset that were also used for the generation of the training mosaics.

Figure 7a presents a test mosaic image containing texture images in its regions that were also used in the training mosaics. As can be seen in Figure 7b, a multi-class pixel labeling convolutional encoder–decoder neural network is able to capture the general mosaic structure if the mosaic has texture classes that were also used in the training set, but it lacks accuracy to precisely determine the borders between two or more different texture regions. Also, inside texture regions there are pixels that are wrongly labeled. This is the reason most segmentation techniques employ a post-processing algorithm to eliminate those small regions and obtain a well-defined image. The HED architecture is also able to capture the general structure of the borders between the different texture regions, as shown in Figure 7c, but due to its greater complexity, it presents difficulty in precisely finding the edges between textures. Finally, in Figure 7d it is shown that the presented method is able to determine the separation between the different regions with greater precision than in Figure 7c,d.

**Figure 8:**
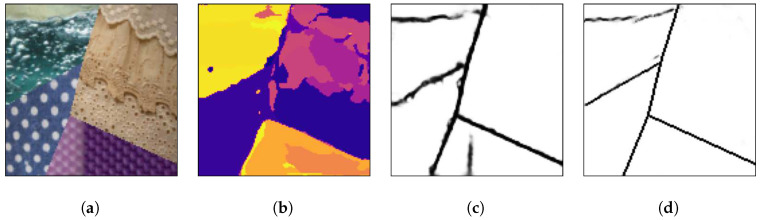
(**a**) An example mosaic, (**b**) the results of a multi-class labeling approach, (**c**) a HED architecture, and (**d**) the proposed method.

**Figure 9:**
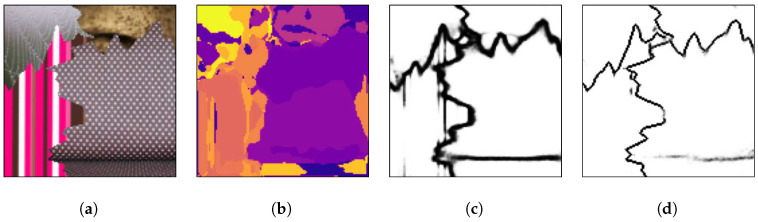
(**a**) An example mosaic, (**b**) the results of a multi-class labeling approach, (**c**) a HED architecture, and (**d**) the proposed method.

**Figure 10:**
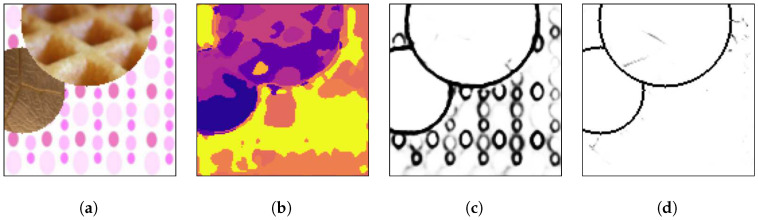
(**a**) An example mosaic, (**b**) the results of a multi-class labeling approach, (**c**) a HED architecture, and (**d**) the proposed method.

Figure 8, Figure 9 and Figure 10 show random mosaics from the fourth test set, which has textures from the Describable Textures Dataset, and the results from a multi-class approach, a HED architecture, and the presented technique.

Figure 8b, Figure 9b and Figure 10b show the results for the multi-class pixel labeling approach when texture classes that were not present in the training set are used in the mosaics. The network is not able to capture the general mosaic structure with the same performance when compared to Figure 7b, where there were known classes in the mosaic regions, wrongly classifying many pixels in an internal texture region where only a single label should be used, especially when the texture class differs in structure and appearance from the texture classes on the training set.

On the other hand, as shown in Figure 8c, Figure 9c and Figure 10c, a HED architecture results in an over-segmentation of the mosaic image due to its complex structure, especially in textures with high internal contrast. In the lower texture region of Figure 8c, there is a contrast between two regions, and the HED architecture is susceptible to it. In the lower-right texture of Figure 9c, there is a sudden change of scale and illumination in the texture region, and the HED architecture is also susceptible to it. It can be seen that this change of scale also affects the presented method, shown in Figure 9d, but with less intensity. Also, the vertical lines of the leftmost texture slightly affects the result from the HED architecture. Finally, in Figure 10c, the small and repeated ellipses are over-segmented by the HED architecture.

The results from the proposed technique are presented in Figure 8d, Figure 9d and Figure 10d. It can be seen that the method precisely identifies the border between different texture regions, as opposed to a multi-class label method, without over-segmenting the internal texture regions, as the HED architecture does. Moreover, the performance of the proposed method remains the same when new texture classes, not used for the training set, are included in the mosaics.

### 4.2. Results on the Different Test Sets

Since the main contribution of this paper is a class-independent texture segmentation method, in order to validate the robustness of the technique, the test was performed on four different sets, each one with 10,000 mosaics. The mosaics of the first test set were created with the 103 texture images from Prague Texture Segmentation Datagenerator and Benchmark dataset used for the training whereas the mosaics of the second test set were created with the remaining 11 texture images from the same dataset that were not used for training the network. The mosaics of the third and fourth set were created with textures from the Brodatz dataset and from the Describable Textures Dataset, respectively, that were also not used for the training.

To quantify the results, the F-measure, the area under the precision–recall curves, and the Pratt Figure of Merit were used to evaluate the performance of the technique. The F-measure is the harmonic mean between precision and recall measures [91], and can be described as:

(2) $$F-\mathrm{measure}=2\frac{\mathrm{precision}\times \mathrm{recall}}{\mathrm{precision}+\mathrm{recall}},$$

where the precision is the measure of correctly classified border pixels relative to all pixels classified as borders, and the recall is the measure of correctly classified border pixels relative to all pixels that should be classified as borders.

A precision–recall curve is a plot of the precision and the recall for different probability thresholds. The F-measure and the precision–recall curves were chosen to evaluate the method performance because of the imbalanced distribution between the two classes, since the number of pixels that belong to internal texture regions is considerably larger than the number of pixels that belong to the borders between textures. Therefore, those metrics are more adequate when dealing with imbalanced datasets [92,93,94].

The Pratt Figure of Merit, *R*, is a measure that balances the errors caused by missing valid edge points, the failure to localize edge points, and the classification of noise fluctuations as edge points [95]. Mathematically, it is described as:

(3) $$R=\frac{1}{\max(I_{I},I_{A})}\sum_{i=1}^{I_{A}}\frac{1}{1+ad^{2}},$$

where $I_{I}$ and $I_{A}$ represent the number of ideal and actual edge map points, respectively, *a* is a scaling constant, usually set to 1/9 [96,97,98], and *d* is the separation of an actual edge point normal to a line of ideal edge points. The measure is normalized so that its value for a perfectly detected edge equals one.

**Figure 11:**
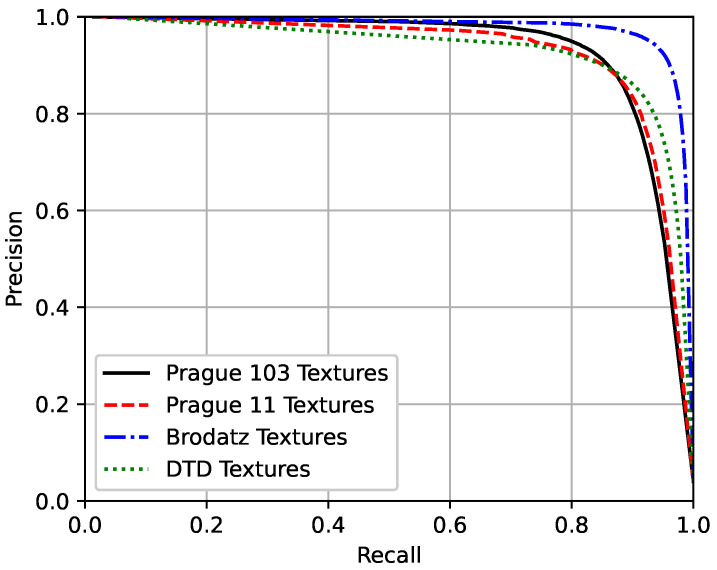
Precision–recall curves for the four different test sets.

**Figure 12:**
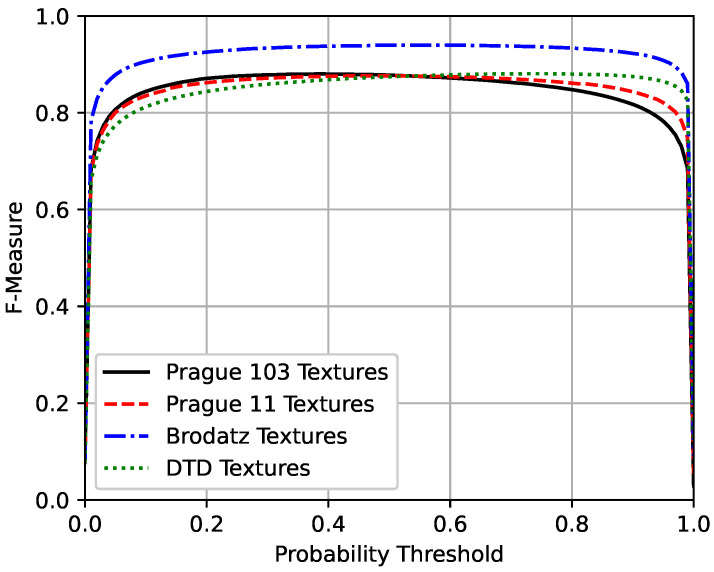
F-measure curves for the four different test sets.

**Figure 13:**
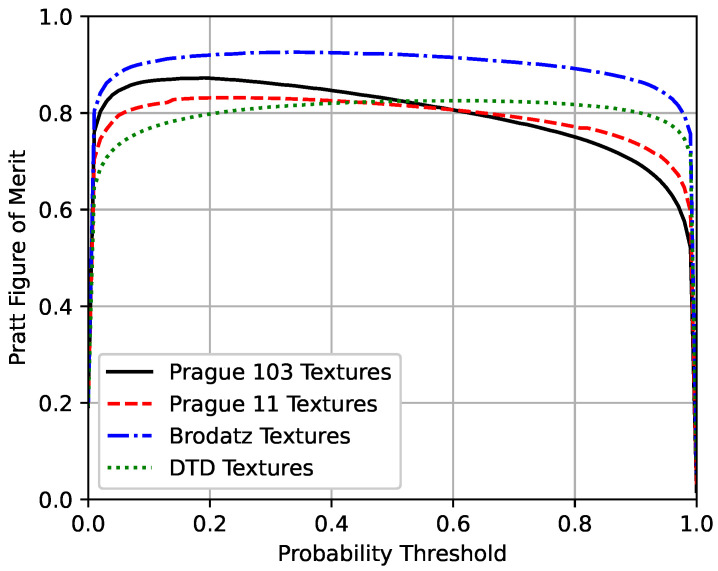
Pratt Figure of Merit curves for the four different test sets.

Figure 11, Figure 12 and Figure 13 present the mean precision–recall curves, the mean F-measure for different probability threshold, and the mean Pratt Figure of Merit for different probability thresholds, respectively, whereas Table 3 presents the area under the mean precision–recall curves, the maximum mean F-measure, and the maximum mean Pratt Figure of Merit achieved for each test set.

Ideally, a perfect model would have the precision and the recall values equal to one for all the different probability thresholds, such that the area under the precision–recall curve and the F-measure would equal the unity. As can be seen in Table 3, the results achieved by the proposed method are close to the unity, and are similar between the four different test sets. Also, the results obtained from the second, third, and fourth datasets, which did not include any textures seen during the training suggest that the model is indeed able to segment mosaic with any texture classes and is not restricted to the classes seen during the training phase.

The Brodatz textures dataset is known to be easy, with remarkable intensity differences between the different classes, so it is expected that the model performs better on this dataset. In addition, it is worth noting that the results between the two Prague textures test sets are close to each other. The DTD dataset is the most challenging one of the used texture sets, but, as seen in Figure 8, Figure 9 and Figure 10, the model was capable of separating the different textures and most of the errors obtained were due to heterogeneous non-repetitive regions inside textures.

### 4.3. Comparison with State-Of-The-Art Methods

Since the training of the presented method was made using textures from the Prague Texture Segmentation Datagenerator and Benchmark, in this subsection, a comparison is made between the edges that results from the presented technique and the results related to be the current best texture segmentation methods when considering this dataset.

**Figure 14:**
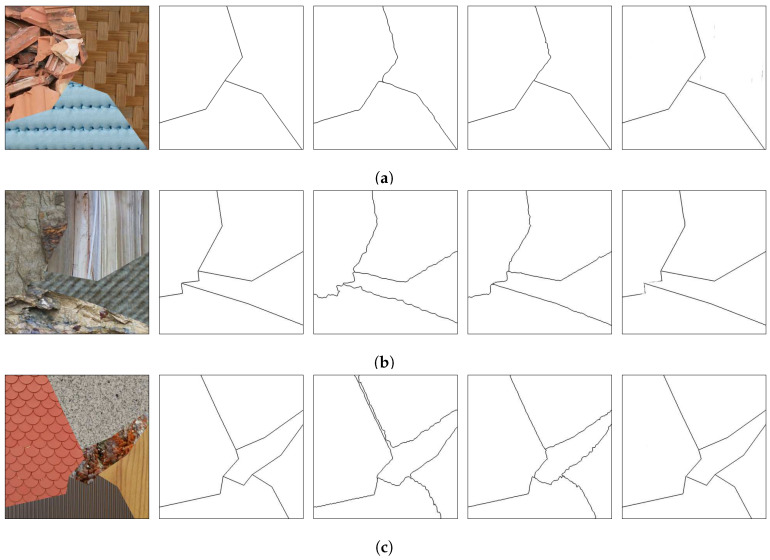
Comparison on the Prague Texture Dataset Generator and Benchmark [70]. From the first to the fifth column, respectively: original images; ground truths; edges produced by the network in [67]; edges produced by the network in [69]; edges produced in the presented method. Letters (**a**–**c**) present different benchmark textures mosaics and are used for referencing the images in the text.

**Figure 15:**
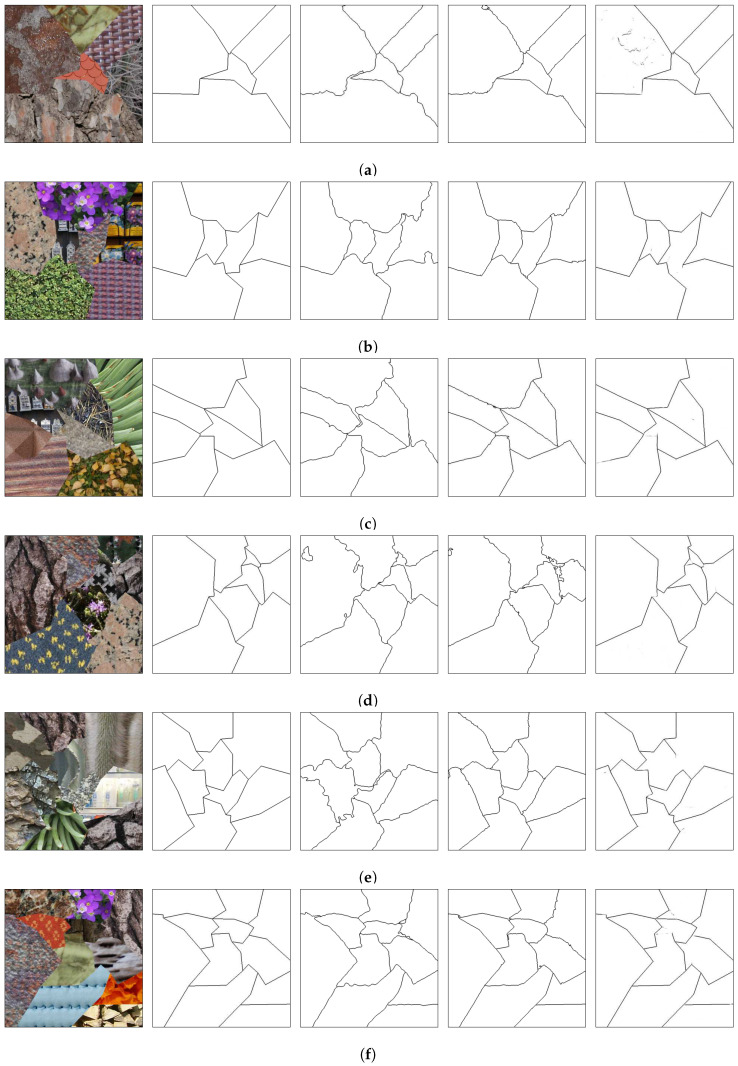
Comparison on the Prague Texture Dataset Generator and Benchmark [70]. From the first to the fifth column, respectively: original images; ground truths; edges produced by the network in [67]; edges produced by the network in [69]; edges produced in the presented method. Letters (**a**–**f**) present different benchmark textures mosaics and are used for referencing the images in the text.

**Figure 16:**
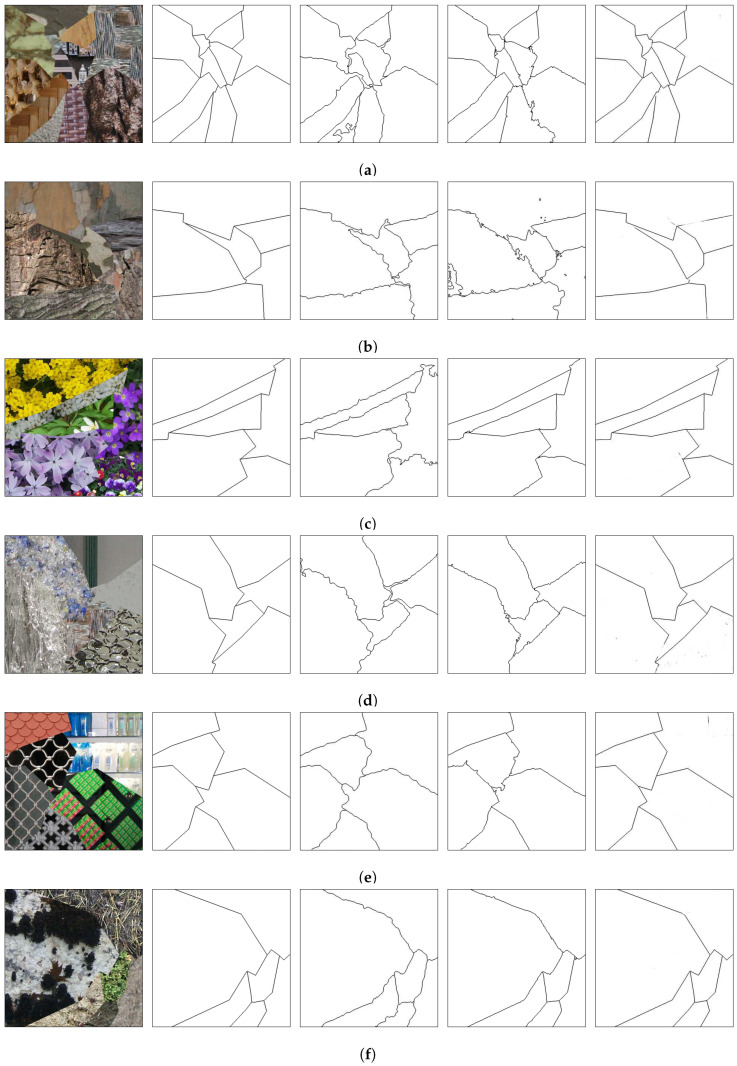
Comparison on the Prague Texture Dataset Generator and Benchmark [70]. From the first to the fifth column, respectively: original images; ground truths; edges produced by the network in [67]; edges produced by the network in [69]; edges produced in the presented method (continuation). Letters (**a**–**f**) present different benchmark textures mosaics and are used for referencing the images in the text.

**Figure 17:**
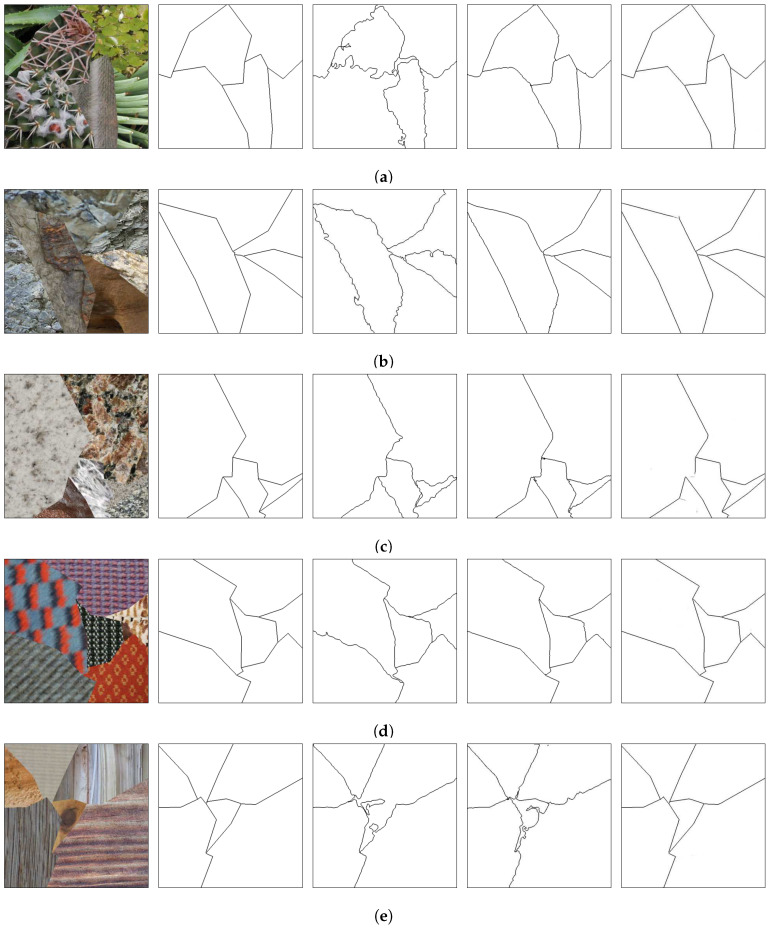
Comparison on the Prague Texture Dataset Generator and Benchmark [70]. From the first to the fifth column, respectively: original images; ground truths; edges produced by the network in [67]; edges produced by the network in [69]; edges produced in the presented method (continuation). Letters (**a**–**e**) present different benchmark textures mosaics and are used for referencing the images in the text.

The best texture segmentation results are the ones from the method presented by Andrearczyk and Whelan [67], and the ones from the method presented by Huang et al. [69]. Those methods use a multi-class pixel labeling approach followed by a post-processing step to clean the regions by eliminating the small ones. For comparison, the edges between the regions generated by those methods were obtained and are shown in Figure 14, Figure 15, Figure 16 and Figure 17 along with the original mosaics and their respective ground truths, as well as the borders from the presented method.

The presented technique, as seen in the results, generally identifies the edges between the texture regions. Moreover, it does not need either to pre-process the mosaic image (as in [69]) or post-process the images (as in both [67,69]). In terms of network parameters, the technique presented in this paper has only 1,138,081 of them. On the other hand, the network presented in [67] has an order of 70 million parameters whereas the network used in [69] uses an even greater number of parameters, due to the preprocessed features.

It should be noted, however, that the proposed technique has a disadvantage, when compared to the techniques in [67,69], if the pixel regions must be precisely obtained. If neighboring regions have similar textures, the border between the two regions may not be complete or even be absent. This is the case in the upper-right border of Figure 15e and the border on the bottom-center of Figure 16d. In this case, the present method is unable to identify two distinct regions, merging them into one single region.

Table 4 presents the F-measure and the Pratt Figure of Merit for the benchmark images shown in Figure 7, Figure 8, Figure 9 and Figure 10 obtained using the proposed technique, the method presented in [67], and the method presented in [69], in the order that they are presented in those figures. To fairly compare the proposed technique with the other two methods, the threshold value was not optimized, so that the probability used to threshold the borders in the proposed method was 50%.

As seen in Table 4, the proposed technique outperforms the other two methods when identifying the borders between the different mosaic regions.

### 4.4. Results on Remote Sensing Texture Mosaics

In this subsection, the presented technique will be applied to mosaics comprising regions made of remote sensing images from the GeoEye RGB images. The images are also available in the Prague Texture Segmentation Datagenerator and Benchmark [99]. It should be noted however, that as the mosaic structure presented in [99] the mosaics created for this paper only approximately correspond to satellite scenes.

**Figure 18:**
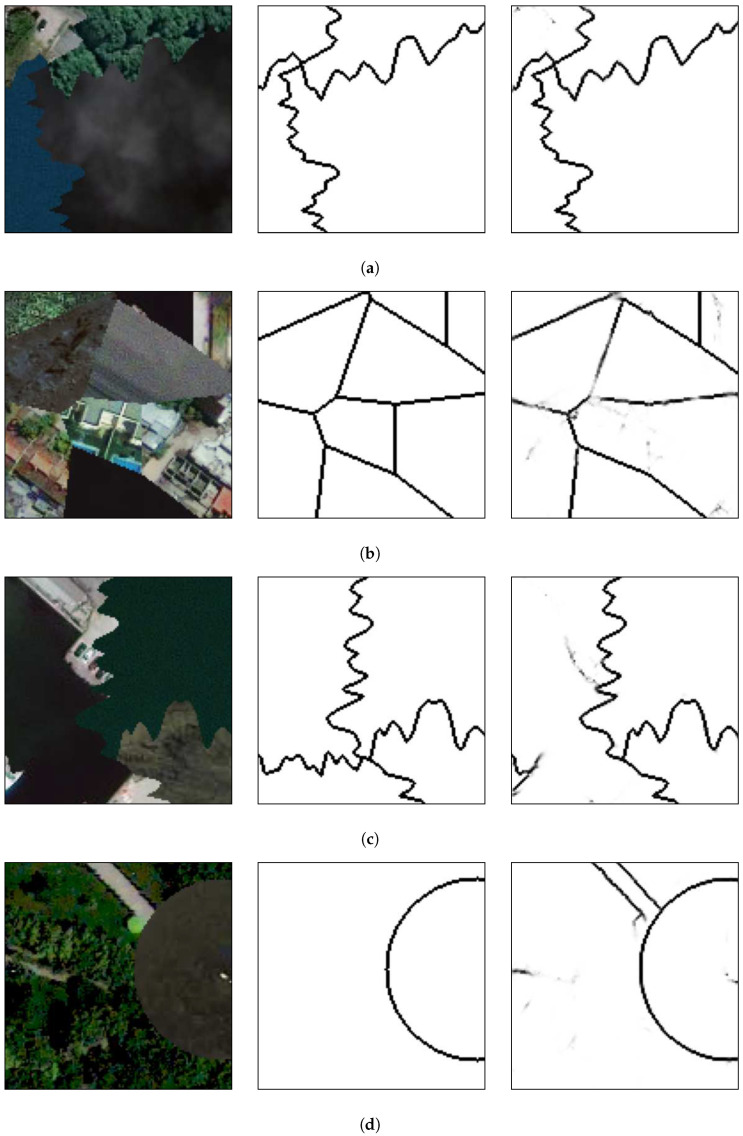
Application on remote sensing images. From left to right, respectively: original image; ground-truth; and the results on the proposed method. Letters (**a**–**d**) present different mosaic images and are used for referencing the images in the text.

Figure 18 presents the results of the methods applied on some of those mosaics. It is worth stating that the network was not trained again using remote sensing images, but it was used directly after being trained on the 103 textures from the Prague Texture Datagenerator and Benchmark.

The proposed method has no difficulty in separating regions when they differ in color and texture characteristics as shown in Figure 18a. In Figure 18b it can be seen that the method could not separate the regions composed of textures from a city landscape, from the center to the right regions. Although those regions were formed from different images, they belong to the same semantic class and even the human eye cannot immediately recognize the separation between these textures. Another difficulty occurs in the bottom of Figure 18c where the texture and color of the two adjacent regions are almost indistinguishable, even to the human eye. Also, the brighter part in the bottom-left part of the image does not present any similarity with the rest of its ground-truth region and is thus separated. Finally, in Figure 18d, the road between the grass does not present any repetitive pattern with other parts of this region and is classified as a separated region.

To quantify the results, 10,000 mosaic images were generated using remote sensing images from the Prague Texture Segmentation Datagenerator and Benchmark [99]. The area under the mean precision–recall curve, the maximum mean F-measure, considering all images, and the maximum mean Pratt Figure of Merit, also considering all images, are presented in Table 5.

As shown in Table 5, due to the fact that remote sensing images have more contextual information rather than solely texture and color characteristics, the observed results have a worse performance when compared with texture mosaics.

**Figure 19:**
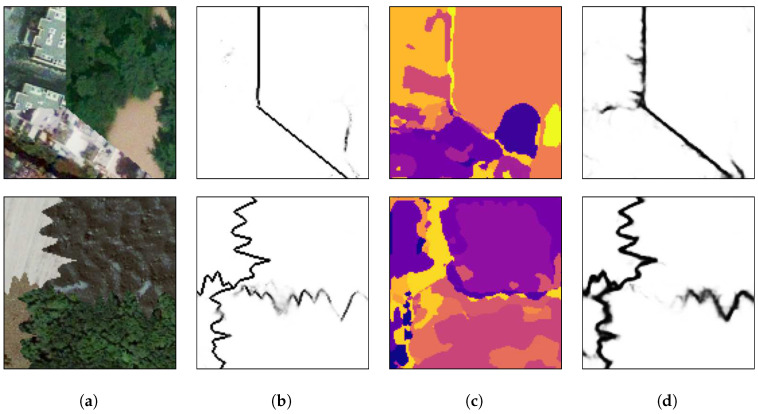
(**a**) An example mosaic, (**b**) the results of a multi-class labeling approach, (**c**) a HED architecture, and (**d**) the proposed method.

For a visual comparison with a multi-class approach and a HED architecture, as presented in Section 4.1, Figure 19 presents additional texture mosaics and the results from the method presented in this paper and the other two approaches. It can be seen that the multi-label method presents difficulties in precisely defining the borders between the regions and that the HED architecture, due to its complexity, has a tendency to excessive segmentation, while the presented method identify the borders with more precision compared to those other approaches.

### 4.5. Results on Tissue Texture Mosaics

In this subsection the present method will be applied on mosaics composed by Hematoxylin and Eosin-stained tissue with malignant lymphoma images [100]. There are three types of malignant lymphoma: chronic lymphocytic leukemia (CLL), follicular lymphoma (FL), and mantle cell lymphoma (MCL). This way, the mosaics were formed by a maximum of three different regions, and each region was randomly filled with a tissue images of one of those three types of malignant lymphoma.

**Figure 20:**
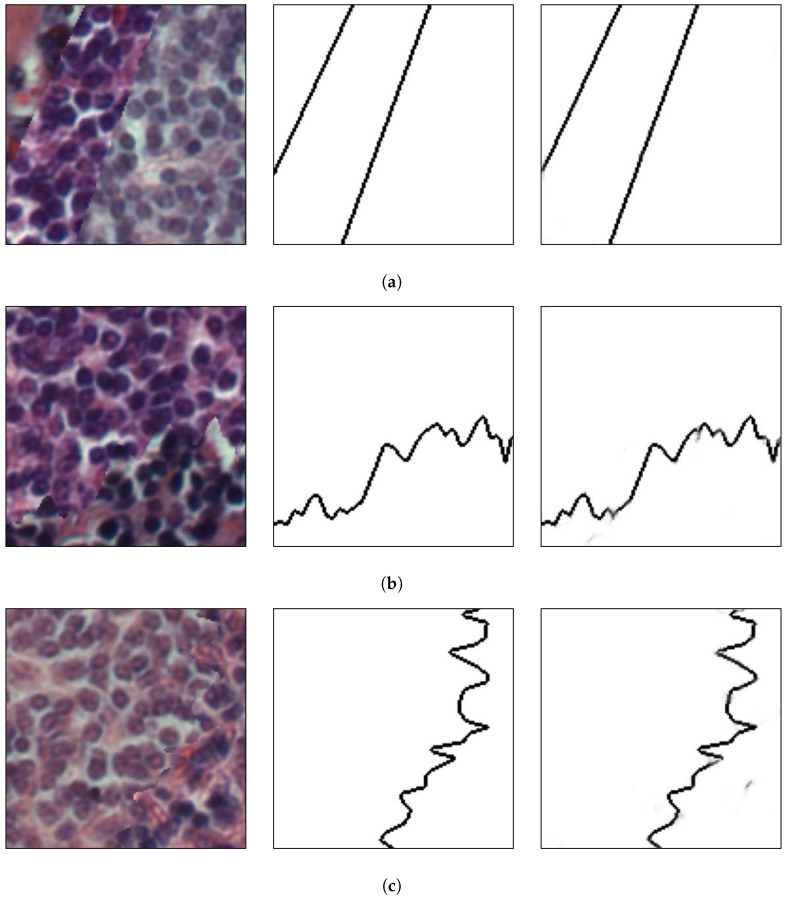
Application on lymphoma tissue images. From left to right, respectively: original image; ground-truth; and the results on the proposed method. Letters (**a**–**c**) present different mosaic images and are used for referencing the images in the text.

It is worth stating that the mosaics do not represent real images and were created in order to evaluate the performance of the proposed method on different types of images for various applications. Moreover, the network was applied without any additional training. Figure 20 presents the results of the method on different sample mosaics.

It can be seen that the method could precisely separate the different parts of the image even when they presented similar colors, which is the case for Figure 20b,c. Those results corroborate the robustness of the presented method and its independence of a limited number of texture classes.

As in Section 4.4, to quantify the results, 10,000 mosaic images were generated using H&E-stained tissue images. The area under the mean precision–recall curve, the maximum mean F-measure, considering all images, and the maximum mean Pratt Figure of Merit, also considering all images, are presented in Table 6. The precision and recall measures for this maximum mean F-measure are also presented.

The results in Table 6 show that the presented method is effective in separating regions of H&E-stained tissue images mosaics. This is because texture and color are the main information of those images. Moreover, for a given type of lymphoma malignant tissue, the internal variability inside a mosaic region is rarely observed.

**Figure 21:**
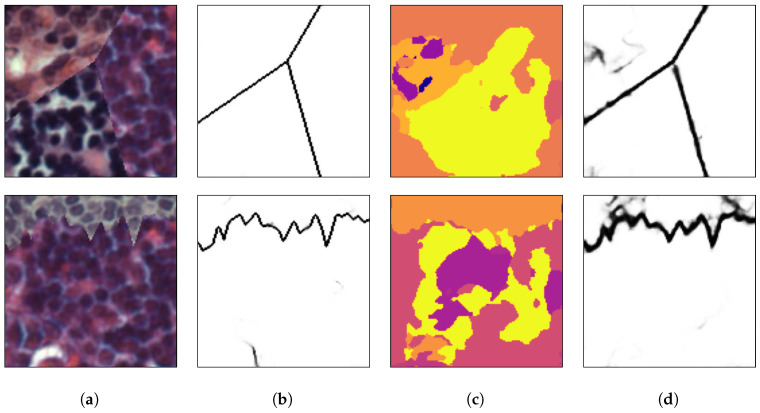
(**a**) An example mosaic, (**b**) the results of a multi-class labeling approach, (**c**) a HED architecture, and (**d**) the proposed method.

For a visual comparison, Figure 21 presents additional texture mosaics and the results from the method presented in this paper and the multi-class approach and the HED architecture. It can be seen that the same characteristics observed in Section 4.1 are present in the mosaics shown.

## 5. Conclusions

This paper proposed a class-independent technique for texture segmentation, thus it is not restricted to a limited number of different texture classes in the image. The segmentation is achieved through a binary pixel-wise classification where each pixel in the image is classified as either belonging to an internal texture region of the image or to a border between two or more textures. The classification is made with a convolutional neural network with an encoder–decoder architecture.

The results suggest that the proposed method is more robust than a multi-class pixel labeling approach, especially in the borders between different textures while it does not over-segment internal texture regions of the image. It is also shown that the technique has a good performance with similar results when considering texture image sets that were not used in the training of the network. The presented technique is also adaptable to different applications and can separate various regions of mosaic images with a smaller number of parameters than similar methods.

## Figures and Tables

**Table 1:** Network architecture parameters.

Number	Layer	Number of Filters
01	Convolution 3 × 3	32
02	Convolution 3 × 3	32
03	Convolution 3 × 3	32
04	Batch Normalization + ReLU Activation	
05	MaxPooling	
06	Convolution 3 × 3	64
07	Convolution 3 × 3	64
08	Convolution 3 × 3	64
09	Batch Normalization + ReLU Activation	
10	MaxPooling	
11	Convolution 3 × 3	128
12	Convolution 3 × 3	128
13	Convolution 3 × 3	128
14	Batch Normalization + ReLU Activation	
15	MaxPooling	
16	Convolution 3 × 3	128
17	Convolution 3 × 3	128
18	Convolution 3 × 3	128
19	Batch Normalization + ReLU Activation	
20	Bilinear Upsampling	
21	Sum (outputs from layers 20 and 14)	
22	Convolution 3 × 3	64
23	Convolution 3 × 3	64
24	Convolution 3 × 3	64
25	Batch Normalization + ReLU Activation	
26	Bilinear Upsampling	
27	Sum (outputs from layers 26 and 09)	
28	Convolution 3 × 3	32
29	Convolution 3 × 3	32
30	Convolution 3 × 3	32
31	Batch Normalization + ReLU Activation	
32	Bilinear Upsampling	
33	Sum (outputs from layers 32 and 04)	
34	Convolution 3 × 3	32
35	Convolution 3 × 3	32
36	Convolution 3 × 3	32
37	Batch Normalization + ReLU Activation	
38	Convolution 3 × 3	1

**Table 2:** Uniform distributions limits used in the image augmentation process.

Procedure	Inferior Limit	Superior Limit
Rotation	0°	360°
Translation	−12 pixels	+12 pixels
Scale change	0.5×	1.5×
Shear	−30°	+30°
Uniform noise	0	0.02
Gaussian smoothing σ value	0	5
Gamma correction γ value	0.5	1.5

**Table 3:** Area under mean precision–recall curve, maximum mean F-measures, and maximum mean Pratt Figure of Merit. The values are expressed as percentage values.

Test Set	Area under Precision–Recall Curve	Maximum F-Measure	Maximum Pratt Figure of Merit
Prague 103 textures	91.690	88.023	87.272
Prague 11 textures	89.960	87.633	83.166
Brodatz textures	92.869	93.996	92.593
DTD textures	86.522	88.071	82.535

**Table 4:** F-measure and Pratt Figure of Merit for the benchmark images from the Prague Texture Datagenerator and Benchmark [70]. The measures are expressed as percentage values. The best results for each image are highlighted in bold.

Image Number	Proposed Technique		Andrearczyk and Whelan		Huang et al.	
	F-Measure	Pratt Figure of Merit	F-Measure	Pratt Figure of Merit	F-Measure	Pratt Figure of Merit
01	**99.9983**	**99.9973**	99.5517	99.9446	99.9207	99.9881
02	**99.9501**	**99.9930**	99.1007	99.9012	99.6143	99.9606
03	**99.9958**	**99.9995**	99.0568	99.6746	99.4385	99.8868
04	**99.9483**	**99.9267**	99.1682	99.8339	99.3949	99.8676
05	**99.9602**	**99.9933**	98.7681	99.7680	99.4474	99.9441
06	**99.9380**	**99.9911**	98.7147	99.7864	99.5670	99.9224
07	**99.9551**	**99.9937**	98.6946	99.6323	99.0139	99.6561
08	**99.9270**	**99.9889**	98.4671	99.5851	99.1441	99.8715
09	**99.9555**	**99.9923**	98.4910	99.7344	99.4847	99.8817
10	**99.9648**	**99.9959**	98.1720	99.4887	99.1403	99.6654
11	**99.9125**	**99.9866**	98.8090	99.7117	98.6177	99.2335
12	**99.9870**	**99.9932**	98.5885	99.6422	99.6217	99.9536
13	**99.9510**	**99.9939**	98.5994	99.6237	99.1777	99.8007
14	**99.9842**	**99.9801**	99.0517	99.8547	99.4689	99.8413
15	**99.9850**	**99.9970**	98.9852	99.8438	99.6247	99.9360
16	**99.9884**	**99.9930**	98.6254	99.3674	99.5489	99.9466
17	**99.9658**	**99.9954**	98.7843	99.7094	99.4507	99.9448
18	**99.9570**	**99.9944**	99.1690	99.8263	99.6912	99.9221
19	**99.9967**	**99.9978**	99.1172	99.8461	99.7941	99.9789
20	**99.9940**	**99.9983**	99.0665	99.7099	99.1944	99.6728

**Table 5:** Area under mean precision–recall curve, maximum mean F-measures, and maximum mean Pratt Figure of Merit for the remote sensing images mosaics. The values are expressed as percentage values.

Metric	Percentage Value
Area under precision–recall curve	84.312
Maximum F-measure	80.063
Maximum Pratt Figure of Merit	76.817

**Table 6:** Area under mean precision–recall curve, maximum mean F-measures, precision and recall for the F-measure presented, and maximum mean Pratt Figure of Merit for the H&E-stained tissue images mosaics. The values are expressed as percentage values.

Metric	Percentage Value
Area under precision–recall curve	98.302
Maximum F-measure	94.872
Precision for the maximum F-measure value	92.757
Recall for the maximum F-measure value	96.488
Maximum Pratt Figure of Merit	92.280

## Abbreviations

The following abbreviations are used in this manuscript:

BSDS	Berkeley Segmentation Dataset and Benchmark
CLL	Chronic lymphocytic leukemia
CNN	Convolutional neural network
DTD	Describable Textures Dataset
FL	Follicular lymphoma
FN	False negatives
FP	False positives
FV	Fisher vector
FCN	Fully convolutional network
HED	Holistically nested edge detector
LBP	Local binary patterns
LSTM	Long short-term memory
MCL	Mantle cell lymphoma
ReLU	Rectified linear unit
SVM	Support vector machine
TN	True negatives
TP	True positives
